# Supplementary material for: Strain versus Tunable Terahertz Nanogap Width: A Simple Formula and a Trench below
Source: Nanomaterials (Basel). 2023 Sep 9;13(18):2526. doi: 10.3390/nano13182526 (PMC10537752; doi:10.3390/nano13182526)
Supplement: Supplementary file 1 [file nanomaterials-13-02526-s001.zip › nanomaterials-2563061-supplementary.pdf]

Supplementary Materials for

Strain versus Tunable Terahertz Nanogap Width: A Simple Formula and a Trench below

Hwanhee Kim [1], Mahsa Haddadi Moghaddam [1], Zhihao Wang [1], Sunghwan Kim [1], Dukhyung Lee [1], Hyosim Yang [1], Myongsoo Jee [2], Daehwan Park [1] and Dai-Sik Kim [1,*]

[1] Department of Physics, Ulsan National Institute of Science and Technology (UNIST), Ulsan 44919, Republic of Korea; psikvv@unist.ac.kr (H.K.)

[2] Quantum Republic Co., Ltd., Rm 805-6 Bldg 106, UNIST-gil, Eonyang-eup, Ulju-gun, Ulsan 44919, Republic of Korea

* Correspondence: daisikkim@unist.ac.kr

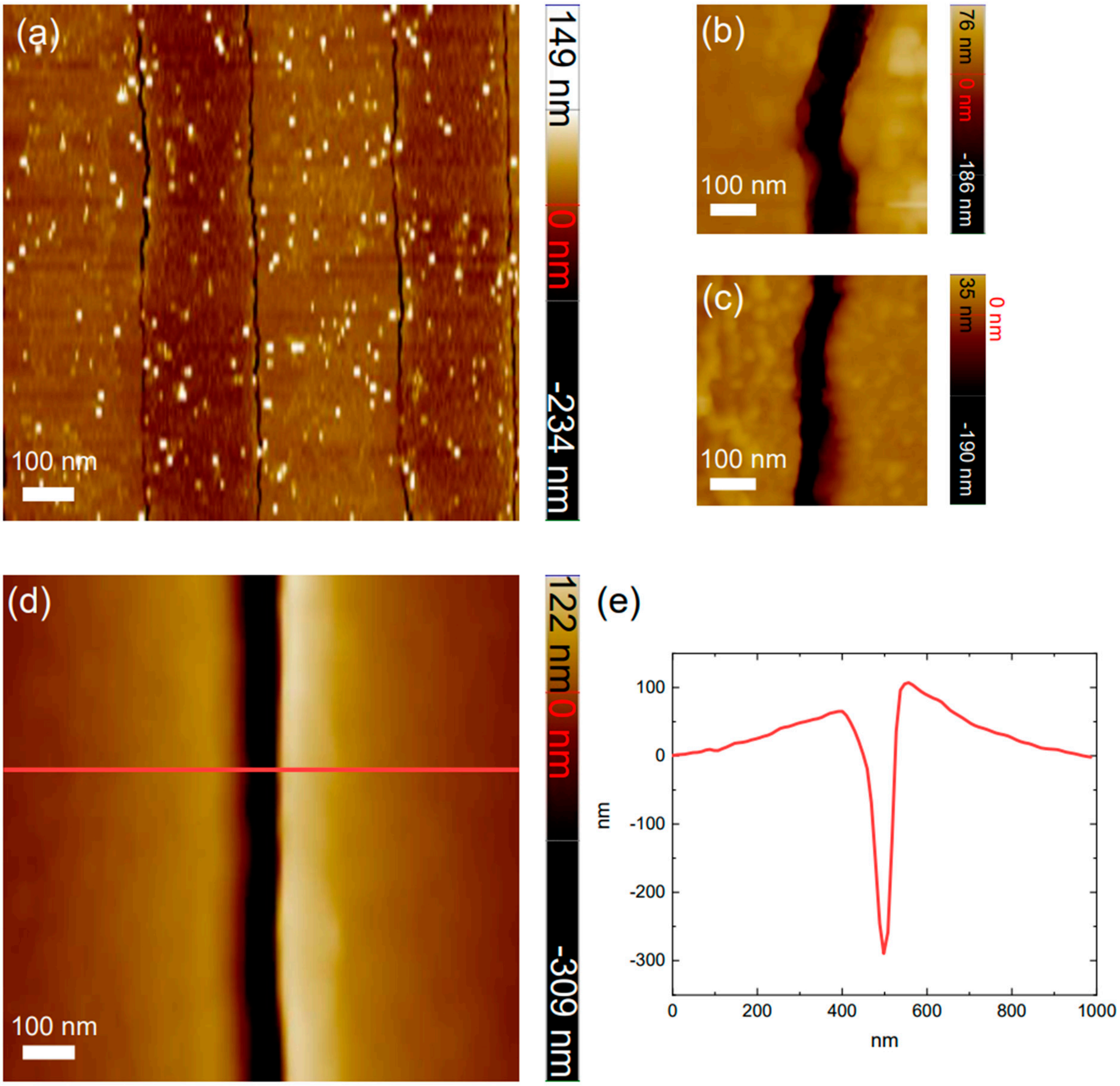

Figure S1. (a) shows the 20 μm × 20 μm size topography zerogap after etching the gold in 0 % strain. (b) is same topography Figure 2 (d). (c) depicts a close-up view of one of the trenches in (a). (d) shows the gold gap topography of the bent zerogap in stage displacement 900 μm (strain = 1.79 %). (e) is the line profile along the red line in (d).
